# Caspase-9 inhibition confers stronger neuronal and vascular protection compared to VEGF neutralization in a mouse model of retinal vein occlusion

**DOI:** 10.3389/fnins.2023.1209527

**Published:** 2023-06-28

**Authors:** Maria I. Avrutsky, Claire W. Chen, Jacqueline M. Lawson, Scott J. Snipas, Guy S. Salvesen, Carol M. Troy

**Affiliations:** ^1^Department of Pathology and Cell Biology, Vagelos College of Physicians and Surgeons, Columbia University, New York, NY, United States; ^2^NCI-Designated Cancer Center, Sanford Burnham Prebys Medical Discovery Institute, La Jolla, CA, United States; ^3^Department of Pathology and Cell Biology, Vagelos College of Physicians and Surgeons, Columbia University, New York, NY, United States; ^4^Department of Neurology, Vagelos College of Physicians and Surgeons, Columbia University, New York, NY, United States; ^5^The Taub Institute for Research on Alzheimer's Disease and the Aging Brain, Vagelos College of Physicians and Surgeons, Columbia University, New York, NY, United States

**Keywords:** retinal vein occlusion, VEGF, caspase-9, cell penetrating peptide, neurodegeneration, neurovascular, edema, ischemia

## Abstract

**Purpose:**

Retinal vein occlusion (RVO) is a sight-threatening condition typically treated with intravitreal injection of vascular endothelial growth factor (VEGF) antagonists. Treatment response to anti-VEGF therapies is highly variable, with poor visual outcomes and treatment response in patients with significant retinal nonperfusion following RVO. Recently, caspase-9 has been identified as a potent regulator of edema, gliosis, and neuronal dysfunction during acute retinal hypoxia. The purpose of this study was to compare the therapeutic effect of caspase-9 inhibition against VEGF-neutralization in an established mouse model of RVO.

**Methods:**

Adult male C57Bl/6 J mice were randomized to induction of RVO and treatment with either vehicle, intravitreal injection of anti-VEGF antibody, topical administration of a selective caspase-9 inhibitor (Pen1-XBir3), or a combination therapy. Animals were followed on days 1, 2, and 8 after RVO with fundus retinal imaging, and with optical coherence tomography (OCT) to capture retinal swelling, capillary nonperfusion (measured by disorganization of retinal inner layers, DRIL), hyperreflective foci (HRF), and retinal atrophy. Focal electroretinography (ERG) measurements were performed on day 7. Histology was performed on retinal sections from day 8.

**Results:**

Both VEGF neutralization and caspase-9 inhibition showed significant retinal protection from RVO compared to vehicle treatment arm. Retinal reperfusion of occluded veins was accelerated in eyes receiving caspase-9 inhibitor, but not significantly different from vehicle in the anti-VEGF group. Retinal edema was suppressed in all treatment groups, with approximately 2-fold greater edema reduction with caspase-9 inhibition compared to VEGF neutralization. HRF were reduced similarly across all treatment groups compared to vehicle. Retinal detachment was reduced only in eyes treated with caspase-9 inhibitor monotherapy. Caspase-9 inhibition reduced retinal atrophy and preserved ERG response; VEGF neutralization did not prevent neurodegeneration following RVO.

**Conclusion:**

Caspase-9 inhibition confers stronger neuronal and vascular protection compared to VEGF neutralization in the mouse laser-induced model of RVO.

## 1. Introduction

Retinal vein occlusion (RVO) occurs when a blockage in one of the major retinal veins obstructs blood outflow from the retina, causing accumulation of fluid, blood, and inflammatory cells. This condition affects between 1 and 2% of persons over the age of 40 (Ho et al., 2016), and can cause variable degrees of vision impairment, depending on the location (macular vs. peripheral), duration, and severity of ischemic injury.

Current treatments for RVO target retinal edema and neuroinflammation via anti-VEGF (vascular endothelial growth factor) treatments, or reduce broad-spectrum inflammation through corticosteroids (Ho et al., 2016). VEGF levels increase in response to hypoxia, and act on endothelial cells to promote vasodilation and increase vascular permeability (Apte et al., 2019). VEGF-neutralizing therapies can be highly effective at resolving retinal swelling and improving visual function, however individual treatment response is variable. Refractory edema persists in over 50% of eyes treated for RVO (Campochiaro et al., 2014), and over 20% of eyes experience visual acuity loss of >15 ETDRS [Early Treatment Diabetic Retinopathy Study] letters over 5  years (Wecker et al., 2017). RVO can damage retinal microvasculature, resulting in poor retinal perfusion and capillary ischemia. Ischemic RVO, typically defined as cases where the ischemic index (percent area of non-perfused retina) is ≥30%, is associated with worse visual outcomes, and poor treatment response (Khayat et al., 2018).

The laser-induced murine model of RVO has been used extensively to investigate mechanisms underlying RVO pathology (Khayat et al., 2017). In this model, an image-guided laser system is used to create a localized occlusion to one or more branch retinal veins. Optimized RVO induction and evaluation protocols enable highly reproducible measurement of retinal edema, inflammation, and neuronal injury (Colón Ortiz et al., 2021; Chen et al., 2022). Consistent with clinical findings, inhibiting VEGF signaling attenuates retinal edema and improves retinal nonperfusion following experimental RVO (Fuma et al., 2017). One novel mediator of retinal injury in RVO is caspase-9, which acts as a multimodal instigator of neurovascular and astroglial dysfunction (Avrutsky et al., 2020; Colon Ortiz et al., 2022). Selective *in vivo* inhibition of caspase-9 can be achieved through administration of Pen1-XBir3 (Akpan et al., 2011; Avrutsky et al., 2020), a formulation containing the caspase-9-inhibitory domain of XIAP protein (BIR3) (Denault et al., 2007) crosslinked to a cell penetrating peptide, Penetratin-1 (Dupont et al., 2011). Typically known for its role as an initiator of the intrinsic apoptosis pathway, caspase-9 mediates diverse inflammatory and degenerative pathologies through both apoptotic and nonapoptotic mechanisms (Avrutsky and Troy, 2021). In RVO, both VEGF and caspase-9 signaling modulate ischemic injury by regulating vascular endothelial cells.

Here, we compared the efficacy of VEGF-neutralization against a cell permeant topical caspase-9 inhibitor (Pen1-XBir3), following induction of RVO. Using an integrated panel of ophthalmic imaging readouts, we evaluated the progression of retinal ischemia, edema, and neurodegeneration in wild-type mice treated with either a VEGF-neutralizing antibody or a topical caspase-9 inhibitor.

## 2. Materials and methods

### 2.1. Randomization and masking

All animals were identified by ear punch, assigned alphanumeric IDs and randomized to treatment groups. RVO induction, retinal imaging, animal exclusions, ERGs, and image analysis was performed by investigators masked to treatment type. Different investigators were responsible for animal randomization, treatment administration, and RVO/retinal imaging procedures.

### 2.2. Animals

Male 2-month old C57Bl6/J mice were purchased from Jackson Laboratories, and allowed to acclimate in specific pathogen-free housing for at least 1 week prior to imaging.

All animals were handled in accordance with the Association for Research in Vision and Ophthalmology (ARVO) statement for the use of animals in ophthalmic and vision research and monitored by the Institutional Animal Care and Use Committee (IACUC) of Columbia University.

### 2.3. RVO procedure

RVO was induced by laser photocoagulation of all major retinal veins (*n* = 3–6 veins occluded/eye) 10–20 min following tail vein injection of Rose Bengal dye (37.5 mg/kg). Animals were anesthetized with intraperitoneal injection of a cocktail of ketamine (80–100 mg/kg) and xylazine (5–10 mg/kg). Eyes were dilated with tropicamide and phenylephrine chloride eye drops. Irradiation of retinal veins was to performed with the 532  nm Micron IV image guided laser system from Phoenix Research Labs by delivering three adjacent laser pulses (power: 100 mW, duration: 1 s, total energy 0.3 J) to each targeted vein at a distance of 375 μm from the optic nerve head. Occlusions were observed by fundus imaging for 1–2 min following laser treatment to record occlusions at Day 0. Exclusion criteria were applied by a masked investigator to identify eyes with fulminant retinal detachment, intravitreal hemorrhage, or reperfusion of all veins within 24 h. Detailed RVO procedure protocols are described in Colón Ortiz et al. (2021).

### 2.4. Anti-VEGF treatment

Animals were anesthetized by intra-peritoneal injection of ketamine (80 mg/kg) and xylazine (5-10 mg/kg), and one drop of 0.5% alcaine was applied to the eye as a topical anesthetic. Sixteen hours prior to RVO mouse anti-VEGF antibody (200µg/ml; R&D Systems) was injected (2 μL) into the vitreous of both eyes using a sterile pulled capillary pipette attached to a Hamilton glass syringe. For controls, eyes were injected with 2 μL of sterile PBS 16 h prior to RVO. After injection, animals received 0.3% topical tobramycin to prevent infection.

### 2.5. Pen1-XBir3 treatment

**Figure 1:**
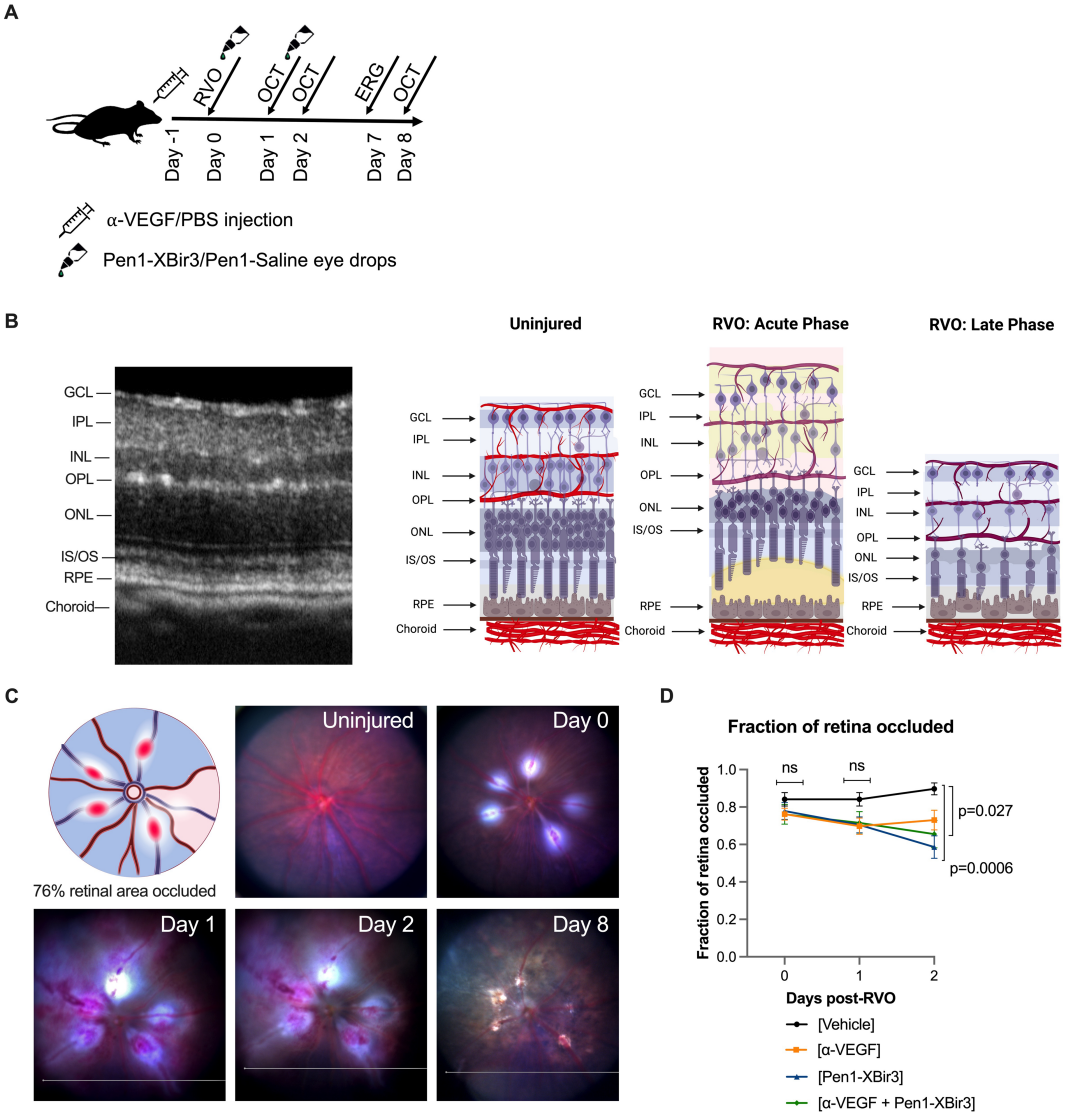
Mouse model of retinal vein occlusion. **(A)** Diagram depicts experimental plan showing timing of treatment administration and retinal imaging in a mouse model of RVO. **(B)** Representative image of an OCT retinal scan labeling retinal layers in an uninjured mouse. Diagram depicts retinal neuronal and vascular layers in uninjured animals and in acute and late phases of RVO. GCL; ganglion cell layer, IPL; inner plexiform layer, INL; inner nuclear layer, OPL; outer plexiform layer, ONL; outer nuclear layer, IS/OS; inner segment/outer segment, RPE; retinal pigment endothelium. Figure made with Biorender.com. **(C)** Fundus retinal imaging of a vehicle treated eye immediately prior to RVO, and at days 0, 1, 2, and 8 post-RVO. Diagram depicts location of occluded veins (red ovals), and shading of retinal occluded area in blue. Figure made with Biorender.com. **(D)** Quantification of the fraction of retina occluded in (*n* = 16–23 eyes/timepoint/group) at days 0, 1, and 2 post-RVO. Graph shows mean ± SEM. Differences between groups measured by Mixed-effects analysis with Tukey’s multiple comparisons test for significance between groups at each timepoint. N eyes/group at 0/1/2 days post-RVO: Vehicle (23/23/19), anti-VEGF (21/21/16), Pen1-XBir3 (18/18/18), anti-VEGF+Pen1-XBir3 (19/19/19).

His-tagged XBir3 was expressed in *Escherichia coli* and purified by nickel column. Pen1 (PolyPeptide Group) was mixed at a 2:1 molar ratio with purified XBir3 and incubated for 1–2 h at 37°C to generate disulfide-linked Pen1-XBir3 as described in Akpan et al. (2011) and Avrutsky et al. (2020). Eye drops containing 10 μg Pen1-XBir3 in sterile saline were administered immediately following RVO, and again at 24 h (Figure 1A). Equivalent volumes of saline containing unlinked Pen1, were administered as controls.

### 2.6. Treatment groups

There were 4 treatment groups. All animals received an injection and eyedrops. The vehicle group received saline injection and Pen1-saline eyedrops (*n* = 23 eyes). Anti-VEGF group received anti-VEGF injection and Pen1-saline eyedrops (*n* = 20 eyes). Pen1-XBir3 group received saline injection and Pen1-XBir3 eyedrops (*n* = 18 eyes). Combination group received anti-VEGF injection and Pen1-XBir3 eyedrops (*n* = 19 eyes).

### 2.7. OCT imaging and analysis

OCT images were captured using the Phoenix Micron IV image-guided OCT system, and layer segmentation was performed using InSight software. Four OCT scans were analyzed from each eye (two vertical and two horizontal scans) positioned approximately 75µm distal from the periphery of RVO burn areas. Intraretinal thickness was defined as GCL through IS/OS, and retinal detachment was quantified as the difference between IS/OS and the RPE (Figure 1B). Fundus images were captured at the time of OCT imaging on days 1, 2, and 8 post-RVO to monitor occlusion resolution following RVO.

HRF counts were measuring using Image J. OCT images were processed using ‘Despeckle’ function. HRF were selected by applying a threshold along the INL selection defined as [mode INL pixel intensity +2 standard deviations], and quantified using ‘Analyze Particles’ function.

DRIL (disorganization of retinal inner layers) was measured as the horizontal extent of each OCT B-scan lacking a distinct boundary between IPL/INL or INL/OPL.

Detailed OCT imaging and analysis protocols for the RVO model are described in (Chen et al., 2022).

### 2.8. ERG imaging

Animals were dark adapted, typically overnight, and subjected to electroretinogram recordings with the Micron IV Image-Guided Focal ERG system. A 1.5 mm flash spot size was centered on the optic nerve head, and a 10 ms white light LED was used to deliver stimulus intensities of −0.7 log (Cd s/m2) and 2.3 log (Cd s/m2). Ten ERG traces were averaged for each eye, and waveforms were analyzed with Labscribe3 ERG to calculate amplitudes of the a wave, b wave and oscillatory potentials (OPs). The sum of the first 6 OPs was used to calculate sum OP amplitude.

### 2.9. Histology

Mice were euthanized with overdose of Ketamine (160–200 mg/kg) plus Xylazine (10–20 mg/kg) and perfused with saline, followed by fixation with 4% paraformaldehyde. Eyes were enucleated, embedded in Optimal Cutting Temperature compound, and cryo-sectioned at 20 μm/section. For H&E staining, slides were submitted to the Columbia University Medical Center Molecular Pathology Shared Resource Histology Service. Ocular sections were imaged with a Nikon microscope (Nikon Instruments) and SPOT digital camera (SPOT Imaging), size bar = 20 μm.

### 2.10. Statistics

Statistical analyses were performed in Graphpad Prism. One-way and two-way ANOVA were used to determine statistical differences between groups. Statistical tests and *p*-values are depicted in figure legends. Data are presented as mean ± SEM. Significance was set to be *p* < 0.05.

## 3. Results

The standard of care for RVO is therapy with injections of anti-VEGF. We have shown that targeting caspase-9 in a mouse model of RVO provides substantial morphologic, cellular and functional protection (Avrutsky et al., 2020; Colon Ortiz et al., 2022). To compare the efficacy of inhibiting caspase-9, vs. blocking VEGF, mice were followed for 8 days following induction of RVO and treatment with either caspase-9 inhibitor eyedrops (Pen1-XBir3), or intravitreal injection of anti-VEGF antibody. We also evaluated a group that received combination therapy to determine if there were additive or potentiating effects of the treatment. Anti-VEGF/PBS administration was performed 16 h prior to induction of RVO to allow for screening out of animals with retinal morphological changes due to the intravitreal injection procedure. Caspase-9 inhibition was achieved by administration of 10 μg Pen1-XBir3 eyedrops immediately following RVO induction, followed by a second dose at 24 h as described in (Avrutsky et al., 2020). RVO-induced retinal pathology was measured by OCT imaging at 1, 2, and 8 days. ERG imaging was performed on dark-adapted animals at 7 days (Figure 1A). Occlusions induced by the laser RVO model typically last through 48 h, and resolve within 1 week. Consequently, the RVO model induces transient retinal detachment and intraretinal swelling characterized by thickening of GCL, IPL, INL and OPL layers at 24–48 h post RVO. By 8 days post-RVO, retinal edema resolves, revealing atrophy of retinal neuronal layers (Figure 1B).

### 3.1. Interventions improve retinal blood flow

Since both caspase-9 inhibition (Avrutsky et al., 2020) and VEGF neutralization (Fuma et al., 2017; Nishinaka et al., 2018) have been associated with improvements in retinal blood flow, we examined the development of retinal ischemia following induction of retinal vein occlusion. Murine retinal vasculature features alternating major retinal veins and arteries that emerge from the optic nerve whose branches dive into the retinal tissues to form three capillary plexi in the GCL, INL, and OPL (Figures 1B,C). The RVO procedure was performed on each major retinal vein, resulting in an average occlusion rate of 77.6% ± 19.2% (mean ± SD) of retinal veins per eye. Retinal occlusion area was defined as the sector of fundus transcribed by the occluded vein and the two adjacent retinal arteries (Figure 1C). There were no significant differences in rates of retinal occlusion immediately after RVO, or at 1 day post-RVO between the treatment groups. However by day 2, we measured significant increase in retinal reperfusion in eyes treated with Pen1-XBir3, either with or without anti-VEGF (Figure 1D). Anti-VEGF treatment on its own was not significantly associated with occlusion resolution.

**Figure 2:**
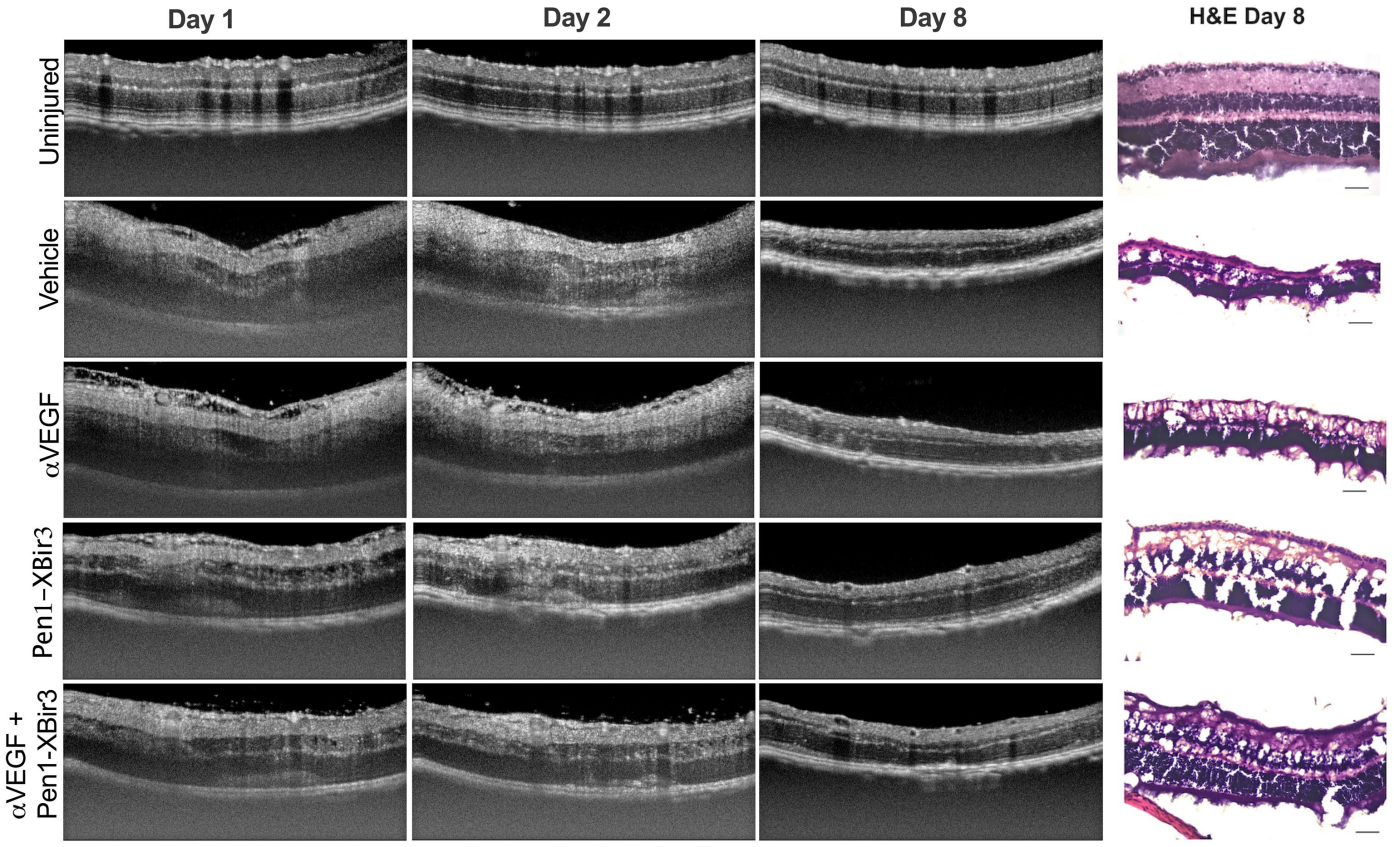
OCT representative images. Figure depicts representative longitudinal OCT scans from an uninjured animal and animals induced with RVO from each treatment group at days 1, 2, and 8. Representative H&E retinal histology sections depicted at right, scale bar = 20 μm.

**Figure 3:**
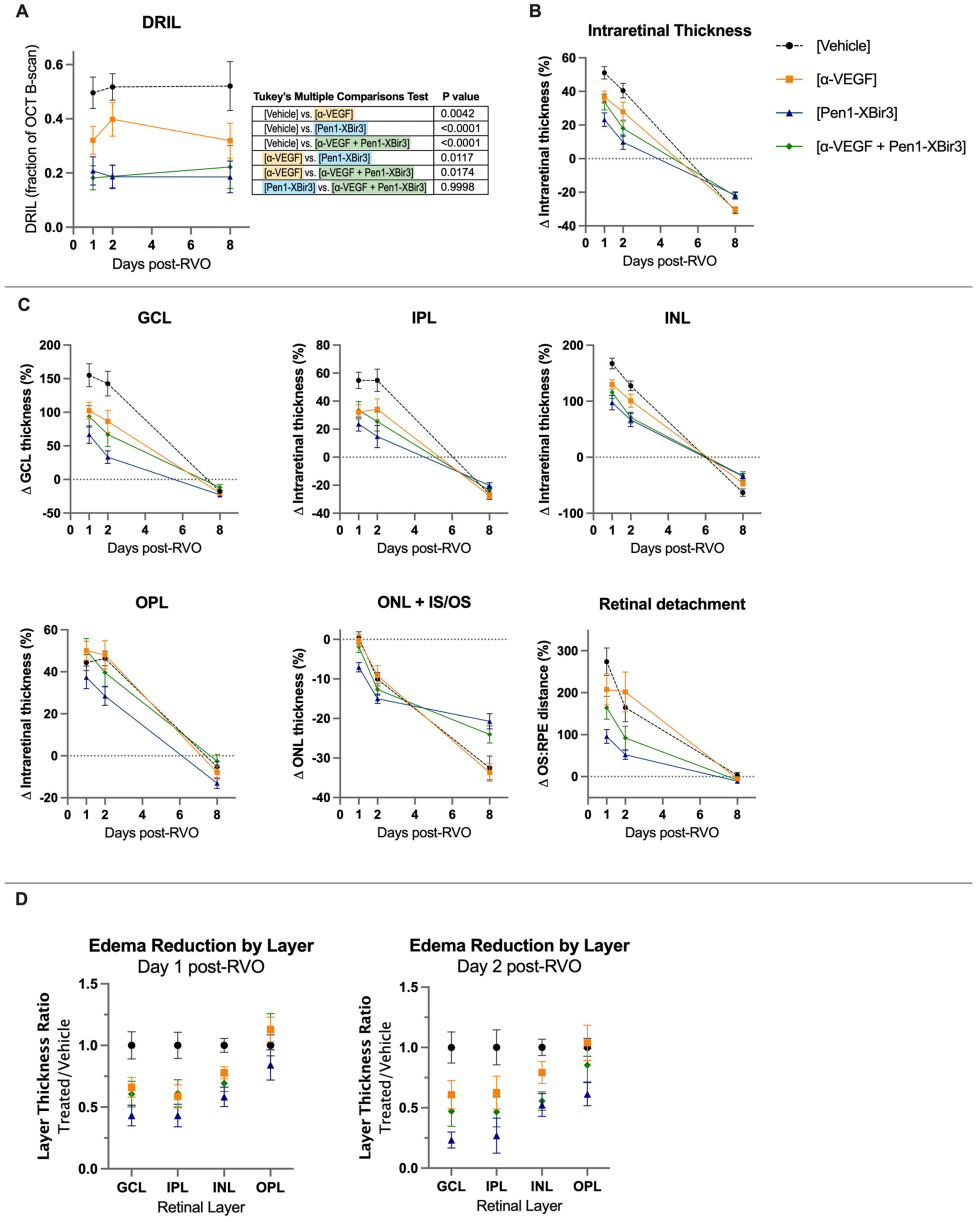
Quantification of DRIL and retinal thickness changes in OCT. **(A)** Quantification of DRIL measurements in (*n* = 16–23 eyes/timepoint/group) each treatment group at days 1, 2, and 8 post-RVO. Graph shows mean ± SEM. Differences between groups measured by 2Way ANOVA with Tukey’s multiple comparisons test to compare main treatment group effect across all study timepoints (days 1/2/8 post-RVO). N eyes/group at 1/2/8 days post-RVO: Vehicle (23/19/18), anti-VEGF (20/16/16), Pen1-XBir3 (18/18/18), anti-VEGF+Pen1-XBir3 (19/19/17). **(B)** Quantification of mean ± SEM change in intraretinal thickness, measured by OCT between the GCL and the IS/OS of (*n* = 16–23 eyes/timepoint/group) at days 1, 2, and 8 post-RVO for each treatment group. Differences between groups at each timepoint compared by 2Way ANOVA with Tukey’s multiple comparisons, statistics shown in Table 1. N at 1/2/8 days post-RVO: Vehicle (23/19/18), anti-VEGF (20/16/16), Pen1-XBir3 (18/18/18), anti-VEGF+Pen1-XBir3 (19/19/17). **(C)** Quantification of mean ± SEM individual retinal layer thickness changes of OCT scans from panel **(B)**. Statistical comparisons from 2Way ANOVA shown in Table 1. **(D)** Quantification of mean ± SEM of retinal thickness changes normalized to RVO/Vehicle treatment group of OCT scans from panels **(B,C)**.

Retinal injury following RVO is significantly associated with microvascular ischemia. To evaluate the evolution of capillary nonperfusion, we analyzed retinal OCTs for DRIL (disorganization of retinal inner layers) (Avrutsky et al., 2020; Chen et al., 2022). DRIL measurements revealed inner retinal nonperfusion across approximately 51% of OCT B-scan length in vehicle treated animals, with no significant temporal trends across the duration of the study (Figures 2, 3A). All treatments were associated with improvement in DRIL, with significantly stronger effect among animals receiving Pen1-XBir3, either as a monotherapy or in conjunction with anti-VEGF.

### 3.2. Caspase-9 and VEGF inhibition regulate retinal edema through partially distinct mechanisms

During the edema phase of RVO, all treatments were associated with less retinal swelling (Figures 2, 3B–D). Representative OCT images are shown for 1, 2, and 8 days; representative H&E histology is shown for 8 days (Figure 2). Anti-VEGF treatment efficacy was most evident in the innermost retinal layers (GCL, IPL, INL), with no measured efficacy on OPL swelling (Figures 3C,D). Eyes treated with Pen1-XBir3 had significantly less swelling in all layers compared to anti-VEGF, and were additionally protected from retinal detachment (Figures 2, 3C; Table 1), however this effect was attenuated in eyes receiving both Pen1-XBir3 and anti-VEGF treatments. Retinal detachment post-RVO is caused by accumulation of subretinal fluid due to dysfunction of the outer blood-retinal barrier. The differential regulation of retinal detachment and intraretinal swelling by Pen1-XBir3 and anti-VEGF treatments suggests that distinct pathophysiological mechanisms may disrupt inner and outer blood retinal barrier in the murine RVO model. Retinal thickness measures in eyes receiving both Pen1-XBir3 and anti-VEGF treatment were not statistically different from either monotherapy, and generally measured an intermediate degree of retinal swelling with no additional benefit over Pen1-XBir3 alone.

**Table 1:** OCT measurement statistics table.

Tukey’s multiple comparisons test	DRIL	Intraretinal thickness	GCL	IPL	INL	OPL	ONL	Retinal detachment
**Day 1**	**Adjusted** ***P*** **Value**	**Adjusted** ***P*** **Value**	**Adjusted** ***P*** **Value**	**Adjusted** ***P*** **Value**	**Adjusted** ***P*** **Value**	**Adjusted** ***P*** **Value**	**Adjusted** ***P*** **Value**	**Adjusted** ***P*** **Value**
[Vehicle] vs. [α-VEGF]	0.1283	0.0401	0.0799	0.028	0.0248	0.768	0.9908	0.5469
[Vehicle] vs. [Pen1-XBir3]	0.0036	<0.0001	0.0012	0.0013	0.0008	0.7206	0.0027	0.0002
[Vehicle] vs. [α-VEGF + Pen1-XBir3]	0.0007	0.0335	0.0602	0.0704	0.0059	0.8103	0.7182	0.0646
[α-VEGF] vs. [Pen1-XBir3]	0.4202	0.0875	0.2116	0.6445	0.183	0.2896	0.0073	0.0467
[α-VEGF] vs. [α-VEGF + Pen1-XBir3]	0.1959	0.9648	0.9734	0.9981	0.7383	>0.9999	0.8762	0.7774
[Pen1-XBir3] vs. [α-VEGF + Pen1-XBir3]	0.9822	0.3492	0.5634	0.5977	0.7134	0.3531	0.0466	0.1717
**Day 2**								
[Vehicle] vs. [α-VEGF]	0.4525	0.3218	0.1277	0.26	0.286	0.9956	0.9908	0.9194
[Vehicle] vs. [Pen1-XBir3]	<0.0001	<0.0001	<0.0001	0.0058	0.0014	0.0161	0.0976	0.0218
[Vehicle] vs. [α-VEGF + Pen1-XBir3]	<0.0001	0.0074	0.0262	0.04	0.0006	0.7932	0.6635	0.3636
[α-VEGF] vs. [Pen1-XBir3]	0.0451	0.0812	0.046	0.2948	0.1939	0.0963	0.1817	0.0325
[α-VEGF] vs. [α-VEGF + Pen1-XBir3]	0.043	0.5671	0.8494	0.8191	0.2211	0.8002	0.6351	0.2194
[Pen1-XBir3] vs. [α-VEGF + Pen1-XBir3]	>0.9999	0.5849	0.3514	0.7333	0.9929	0.4977	0.6055	0.5517
**Day 8**								
[Vehicle] vs. [α-VEGF]	0.29	0.9924	0.8562	0.9932	0.2009	0.9076	0.9891	0.7077
[Vehicle] vs. [Pen1-XBir3]	0.0209	0.0064	0.1077	0.4995	0.0052	0.1373	0.0128	0.2694
[Vehicle] vs. [α-VEGF + Pen1-XBir3]	0.0825	0.0102	0.6044	0.9515	0.0263	0.9062	0.1242	0.4683
[α-VEGF] vs. [Pen1-XBir3]	0.4297	0.0114	0.7913	0.0803	0.2647	0.5697	0.0007	0.4111
[α-VEGF] vs. [α-VEGF + Pen1-XBir3]	0.7782	0.018	0.3736	0.7348	0.4754	0.6221	0.0203	0.8784
[Pen1-XBir3] vs. [α-VEGF + Pen1-XBir3]	0.9819	0.9979	0.1182	0.7368	>0.9999	0.0614	0.6592	0.8501

Green = improvement. Light green = intermediate improvement. White = no significant effect over RVO.

### 3.3. Both caspase-9 inhibition and VEGF neutralization reduce HRF

**Figure 4:**
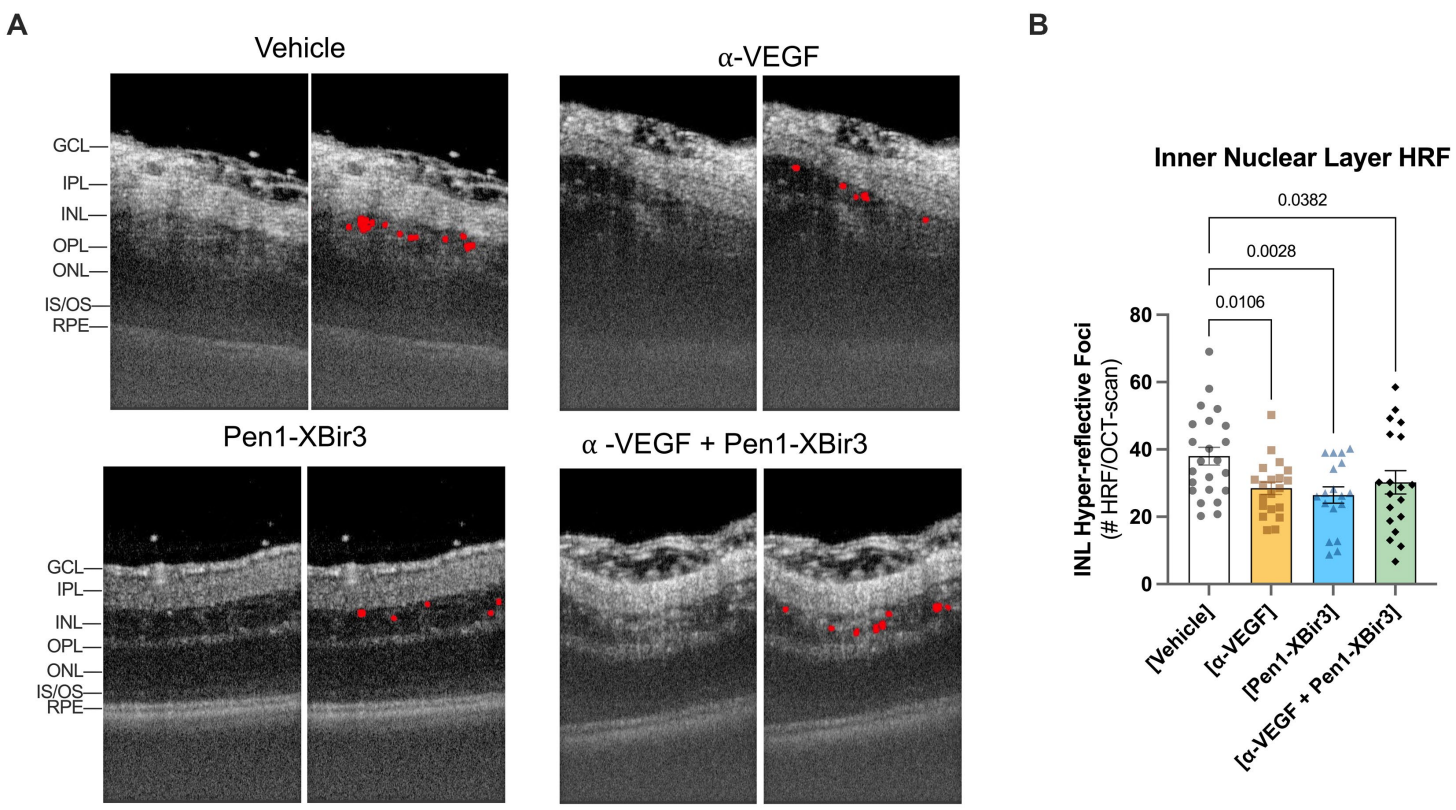
Quantification of hyper-reflective foci (HRF). **(A)** Closeup of representative OCT images at 1 day post-RVO, with overlays showing automated detection of hyperreflective regions. **(B)** Quantification of mean ± SEM number of hyperreflective foci detected in each treatment group at 1 day post-RVO. Differences between groups measured by one-way ANOVA with Fisher’s LSD test. N eyes/group: Vehicle (23), anti-VEGF (20), Pen1-XBir3 (18), anti-VEGF+Pen1-XBir3 (19).

Both in patients and in experimental models of RVO, retinal ischemia is associated with the appearance of hyperreflective foci (HRF) in the inner retina. While the etiology of HRF remains ambiguous, clinical data suggests a strong correlation between HRF, ocular inflammation, and visual prognosis outcomes (Chatziralli et al., 2016; Mo et al., 2017). We evaluated the number of HRFs detected in the INL; all treatments were associated with a 20.5–30.4% decrease in HRF counts compared to vehicle-treated eyes (Figure 4). Unlike the retinal ischemic measurements, there were no significant differences between any of the treatment groups.

### 3.4. Caspase-9 inhibition but not VEGF neutralization, protects against retinal atrophy

At 8 days post-RVO, retinal atrophy was measured in the GCL, IPL, INL, and ONL. Although anti-VEGF treatment effectively attenuated retinal swelling, it was not associated with protection from retinal atrophy (Figures 2, 3B,C). Conversely, both groups that received Pen1-XBir3 had significant protection of INL and ONL layers. The GCL/IPL complex, comprised of retinal ganglion neurons was not significantly protected by either treatment.

**Figure 5:**
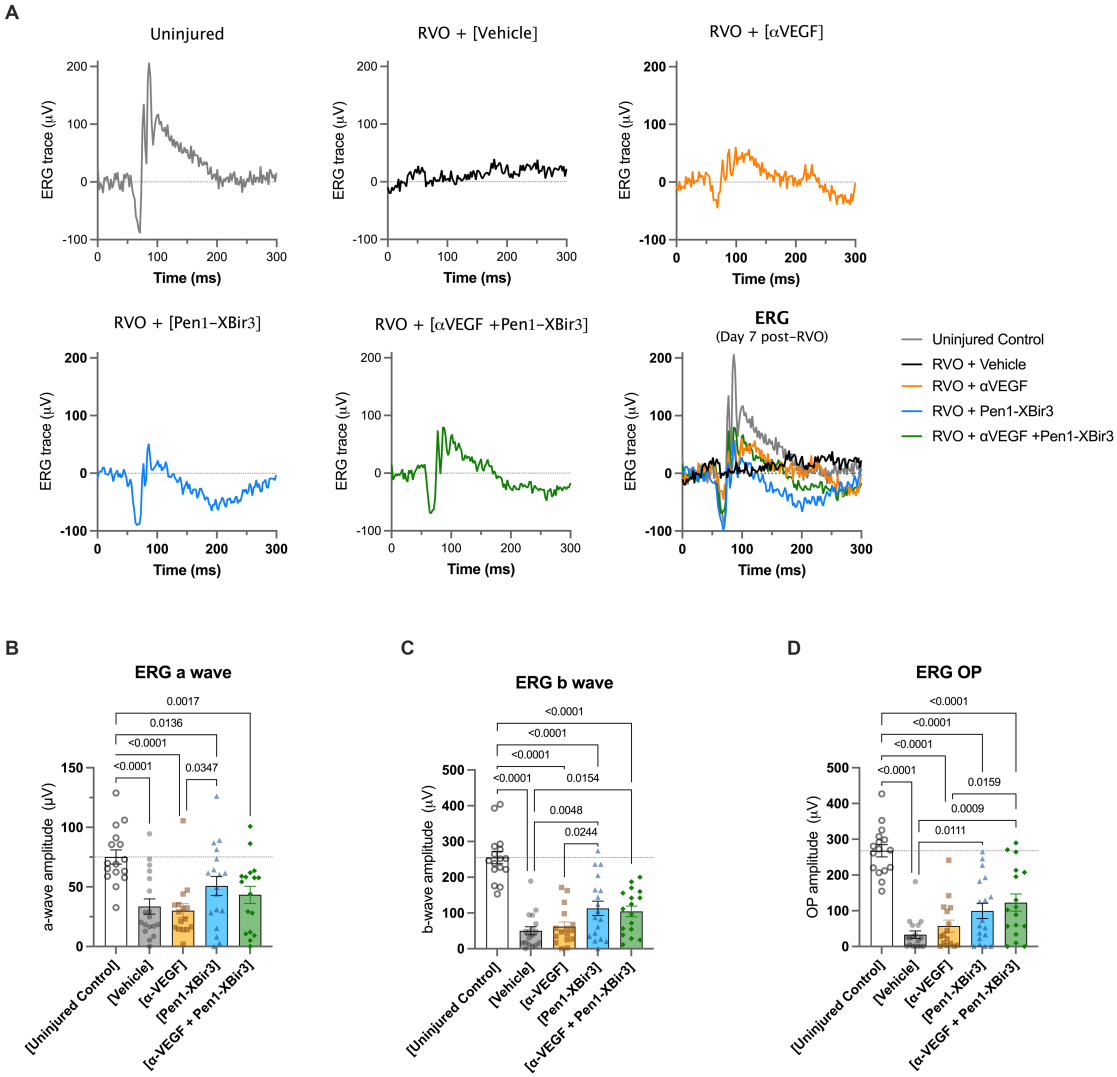
Retinal functional measure by focal ERG. **(A)** Depicts representative electroretinogram (ERG) traces in an uninjured control animal, and in each RVO treatment group. **(B)** Quantification of mean ± SEM a wave amplitudes in (*n* = 16–18 eyes/group) uninjured control mice and in each RVO treatment group. Difference between groups were measured by one-way ANOVA with Fisher’s LSD test. N eyes/group: Vehicle (18), anti-VEGF (16), Pen1-XBir3 (18), anti-VEGF+Pen1-XBir3 (17), uninjured (16). **(C)** Quantification of mean ± SEM b wave amplitudes in (*n* = 16–18 eyes/group) uninjured control mice and in each RVO treatment group. Differences between groups were measured by one-way ANOVA with Fisher’s LSD test. N eyes/group: Vehicle (18), anti-VEGF (16), Pen1-XBir3 (18), anti-VEGF+Pen1-XBir3 (17), uninjured (16). **(D)** Quantification of mean ± SEM OP amplitudes in (*n* = 16–18 eyes/group) uninjured control mice and in each RVO treatment group. Differences between groups were measured by one-way ANOVA with Fisher’s LSD test. N eyes/group: Vehicle (18), anti-VEGF (16), Pen1-XBir3 (18), anti-VEGF+Pen1-XBir3 (17), uninjured (16).

Consistent with OCT measures of retinal atrophy, ERG measurements showed functional neuroprotection with Pen1-XBir3 treatment, but not with anti-VEGF (Figure 5).

## 4. Discussion

This study represents a framework for comparing *in vivo* efficacy of experimental treatments against a clinically-established mechanism of action (VEGF-neutralization) in a mouse model of RVO. Our prior work has shown that non-apoptotic activation of caspase-9 regulates edema, gliosis and neuronal dysfunction in a well-defined mouse model of RVO (Avrutsky et al., 2020; Colon Ortiz et al., 2022). Here, we compare the functional efficacy of Pen1-XBir3, a caspase-9 inhibitor, with an established VEGF neutralizing treatment protocol. The data show that topical treatment with Pen1-XBir3 is equal or superior to intravitreal delivery of VEGF neutralizing antibodies across multiple *in vivo* measures.

Orthogonal measures capture different dimensions of RVO pathology in response to caspase-9 inhibition, VEGF-neutralization, or a combination treatment. Notably, our study was powered to capture therapeutic differences between Pen1-XBir3 treatment versus control, and was not powered to evaluate differences between the combination treatment and either monotherapy. Since neither therapy offers complete retinal protection by itself, a combination treatment was attempted to test the feasibility of using caspase-9 inhibition as an adjunct to VEGF neutralization. To maximize potential efficacy, both treatments were applied at maximal possible dose, based on limitations of intravitreal injection and eye-drop volumes. Stronger protection in eyes treated with the caspase-9 inhibitor suggest that caspase-9 signaling is a more critical target for treating retinal edema and capillary ischemia in RVO. Meanwhile the combination of the two therapies was more effective than anti-VEGF monotherapy, but did not show any improvement over the efficacy of Pen1-XBir3 alone. One limitation of this study is that this was the first attempt to co-administer caspase-9 and VEGF antagonists in a retinal injury model. The dosing levels and methods of delivering the respective treatments were not optimized for a combination therapy approach.

This study captured functional and morphological endpoints, and did not enable molecular profiling of signaling changes in response to the treatment arms. Future studies will be required to determine whether and how caspase-9 and VEGF signaling may interact in RVO. Both caspase-9 and VEGF are multimodal regulators of tissue injury and hypoxia response, with several intersecting mechanisms of action. Both proteins can regulate endothelial cell survival through modulation of autophagy and mitochondrial function (Domigan et al., 2015; An et al., 2019; Spengler et al., 2020). Additionally, both VEGF and caspase-9 have multiple immunomodulatory activities (Reinders et al., 2003; Avrutsky and Troy, 2021).

Using *in vivo* measures over time increases the translational relevance of preclinical models by utilizing techniques which are commonly used in the clinic to diagnose and follow the evolution of disease in persons receiving treatment for RVO. OCT measures retinal thickness, ischemia (using DRIL) and inflammation (tracking HRFs). Fundoscopic imaging follows resolution of retinal occlusions. ERGs reflect integrity of the retinal neuronal network. Efficacy of each treatment arm was equivalent with regard to HRFs but for all other measures Pen1-XBir3 provided superior efficacy compared to VEGF neutralization. Pen1-XBir3 protected neuronal function, while VEGF neutralization did not affect ERG response. These findings are consistent with clinical data noting extensive retinal degeneration after successful resolution of edema with anti-VEGF treatment (Hasegawa et al., 2017), and lack of functional improvements in retinal regions of severe nonperfusion at baseline (Rachima et al., 2020).

## 5. Conclusion

Comparison of VEGF-neutralization and caspase-9 inhibition strategies in a murine model of RVO demonstrate vascular protection by anti-VEGF, and both vascular and neuronal protection with inhibition of caspase-9. These data demonstrate comparison of an experimental therapy against a clinically-validated treatment modality, and support developing therapies to target pathways that are not VEGF-driven for the treatment of RVO.

## Data availability statement

The original contributions presented in the study are included in the article/supplementary material, further inquiries can be directed to the corresponding author.

## Author contributions

CT and MA contributed to the conception and design of the study. MA, CC, and JL performed the experimental procedures and data analysis. SS and GS generated XBir3 protein used in the study. CT, MA, and CC contributed to the writing, revision, and figures. CT acquired the funding. All authors contributed to the article and approved the submitted version.

## Funding

This work was supported by a Sponsored Research Agreement with Opera Therapeutics to CT, by the National Institute of Neurological Disorders and Stroke (RO1 NS081333 to CT) and the Department of Defense Army/Air Force (DURIP to CT) and by the National Eye Institute (T32 EY013933 to MA).

## Conflict of interest

CT and MA are listed as inventors on patent applications filed by the Trustees of Columbia University in the City of New York related to the therapeutic use of caspase-9 inhibitors. MA received consulting income from Opera Therapeutics.

The remaining authors declare that the research was conducted in the absence of any commercial or financial relationships that could be construed as a potential conflict of interest.

## Publisher’s note

All claims expressed in this article are solely those of the authors and do not necessarily represent those of their affiliated organizations, or those of the publisher, the editors and the reviewers. Any product that may be evaluated in this article, or claim that may be made by its manufacturer, is not guaranteed or endorsed by the publisher.
